# The deubiquitinase USP9X regulates FBW7 stability and suppresses colorectal cancer

**DOI:** 10.1172/JCI97325

**Published:** 2018-02-26

**Authors:** Omar M. Khan, Joana Carvalho, Bradley Spencer-Dene, Richard Mitter, David Frith, Ambrosius P. Snijders, Stephen A. Wood, Axel Behrens

**Affiliations:** 1 Adult Stem Cell Laboratory,; 2 Experimental Histopathology,; 3 Bioinformatics and Biostatistics, and; 4 Proteomics, The Francis Crick Institute, London, United Kingdom.; 5 Griffith Institute for Drug Discovery, Griffith University, Nathan, Queensland, Australia.; 6 King’s College London, Faculty of Life Sciences and Medicine, Guy’s Campus, London, United Kingdom.

**Keywords:** Cell Biology, Gastroenterology, Colorectal cancer, Ubiquitin-proteosome system

## Abstract

The tumor suppressor FBW7 targets oncoproteins such as c-MYC for ubiquitylation and is mutated in several human cancers. We noted that in a substantial percentage of colon cancers, FBW7 protein is undetectable despite the presence of *FBW7* mRNA. To understand the molecular mechanism of FBW7 regulation in these cancers, we employed proteomics and identified the deubiquitinase (DUB) USP9X as an FBW7 interactor. USP9X antagonized FBW7 ubiquitylation, and *Usp9x* deletion caused Fbw7 destabilization. Mice lacking *Usp9x* in the gut showed reduced secretory cell differentiation and increased progenitor proliferation, phenocopying *Fbw7* loss. In addition, *Usp9x* inactivation impaired intestinal regeneration and increased tumor burden in colitis-associated intestinal cancer. *c-Myc* heterozygosity abrogated increased progenitor proliferation and tumor burden in *Usp9x*-deficient mice, suggesting that Usp9x suppresses tumor formation by regulating Fbw7 protein stability and thereby reducing c-Myc. Thus, we identify a tumor suppressor mechanism in the mammalian intestine that arises from the posttranslational regulation of FBW7 by USP9X independent of somatic *FBW7* mutations.

## Introduction

The mammalian intestine is composed of repetitive differentiated and stem cell units called villi and crypts, respectively. Stem cells are located at the bottom of crypts, where they produce highly proliferating transit-amplifying (TA) cells. TA cells differentiate into absorptive and secretory cells, the two main intestinal lineages. The absorptive lineage comprises enterocytes, while the secretory lineage is composed of goblet (mucin-secreting), enteroendocrine (hormone-secreting), and Paneth cells (which produce lysozyme) (1). Pathways including Wnt and Notch signaling (2) regulate the proliferation and differentiation of the mammalian gut epithelium. Wnt ligands activate β-catenin and increase expression of Wnt/T cell factor (TCF) target genes (3), including *c-Myc*, which is required for stem cell maintenance (2). Notch signaling is active both in stem cells and in TA cells, where it controls cell fate decisions (4).

The importance of Wnt signaling in gut homeostasis is highlighted by the recurring mutations of the adenomatous polyposis coli (*APC*) gene (5), encoding a negative regulator of Wnt signaling, in human colorectal cancers (CRCs). APC inhibits Wnt signaling by forming a cytosolic destruction complex with GSK3β and AXIN to allow E3 ubiquitin ligase–mediated proteasomal degradation of β-catenin (2). Mutant APC fails to target β-catenin for proteasomal degradation and thus results in increased downstream Wnt signaling, including increased *c-Myc* expression. c-Myc is the main mediator of Wnt/β-catenin function in CRC (6) and is required for formation of intestinal polyps in *Apc^min/+^* mice (7).

In addition to somatic *APC* mutations, chronic inflammatory conditions including Crohn’s disease and ulcerative colitis also predispose patients to CRC (8, 9). Although mechanisms linking inflammatory colitis to CRC are incompletely understood, these cancers may also show activation of Wnt signaling, including activating β-catenin mutations (10), or amplification of *c-MYC* (11).

The tumor suppressor FBW7 functions as the substrate recognition component of an SKP1, CUL1, and F-box protein–type (SCF-type) E3-ubiquitin ligase complex (12) targeting several oncoproteins, including c-MYC, NICD1 (Notch1 intracellular domain), and c-JUN, for degradation (13–22). These proteins have well-defined roles in gut homeostasis and CRC (23–25), and their accumulation induces overproliferation and impaired differentiation of the intestinal epithelium. Consistent with this function, *FBW7* is one of the most frequently mutated genes in human CRC, altered in approximately 10% of tumors (26, 27), and *Fbw7* inactivation accelerates tumorigenesis in *Apc^min/+^* mice (13, 28).

Enzymes counteracting the E3 ligases are the deubiquitinases (DUBs), which act by cleaving the ubiquitin chains off the substrate protein, leading to either protein stabilization or change in its activity (29). Emerging evidence suggests that DUBs are also required for the maintenance of tissue homeostasis. For example, we previously showed that Usp28 deubiquitylates some Fbw7 substrates, thereby regulating murine gut homeostasis (30). In addition, Usp22 promotes CRC by stabilizing cyclin B1 (31). Thus, deregulation of either ubiquitylation or deubiquitylation may result in cancer.

The mechanism of tumor suppression by FBW7 has been intensively studied, and many relevant SCF(FBW7) substrates have been identified. However, the mechanisms controlling FBW7 function have been insufficiently studied. Here we report that FBW7 protein is consistently downregulated in human CRC and identify the DUB USP9X as a positive regulator of FBW7 stability. USP9X directly bound FBW7 and antagonized its ubiquitylation and proteasomal degradation. In murine gut, this positive regulation was required for maintenance of tissue homeostasis mainly via suppression of c-Myc and Notch1 proteins. Colitis-driven tumor formation was greatly accelerated in mice with intestine-specific deletion of *Usp9x*. Using genetic rescue experiments, we show that the increased tumor burden in *Usp9x*-deficient colon was mainly mediated by c-Myc accumulation. In addition, we find a strong correlation between USP9X and FBW7 immunohistochemical staining in human CRC and show that reduced USP9X was strongly associated with poor clinical outcome in those cancers.

## Results

### FBW7 protein is low or absent in most human CRCs.

**Figure 1:**
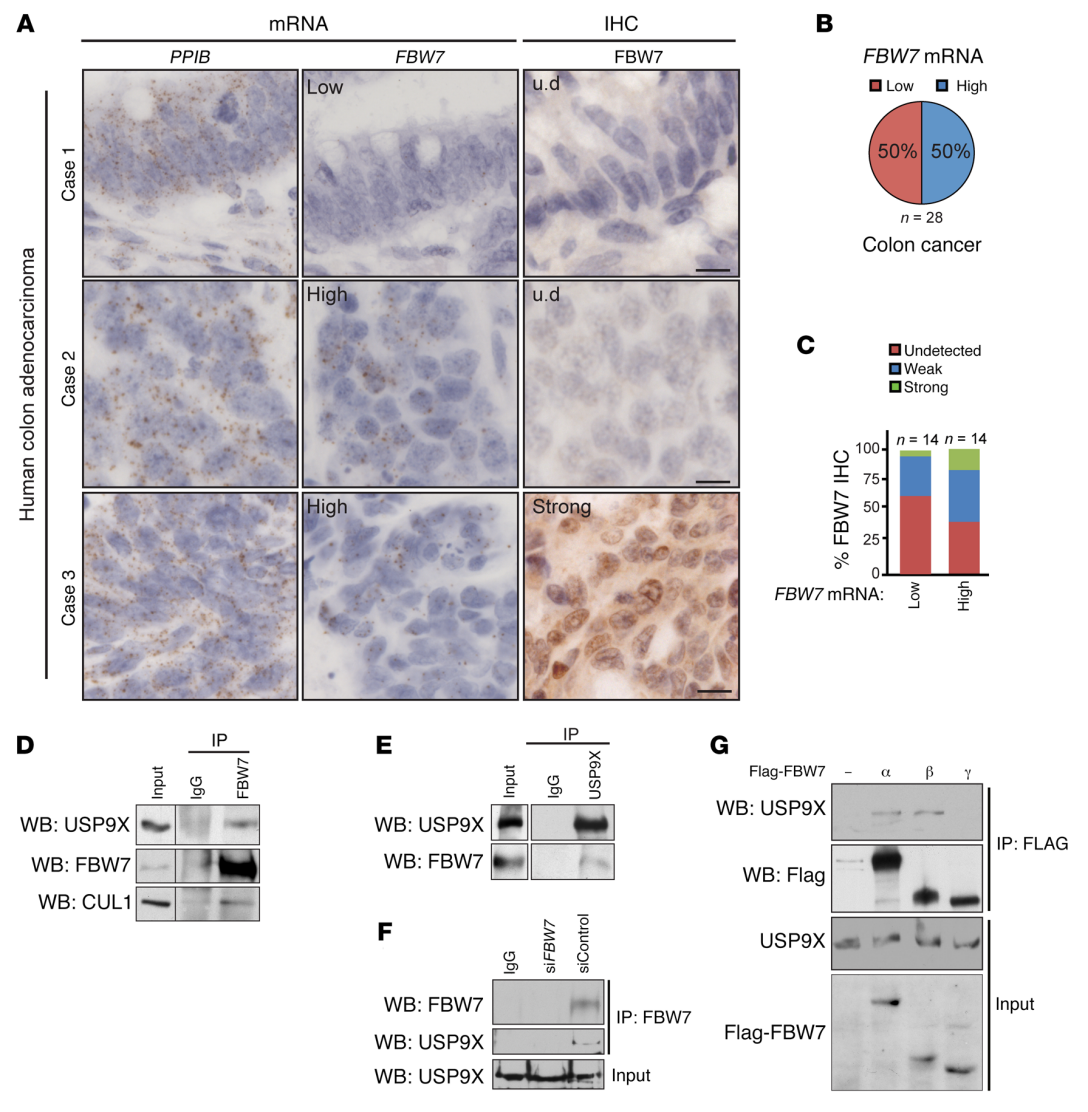
FBW7 is downregulated in human CRC. (**A**) Representative images of RNAscope for *PPIB* and *FBW7*, and FBW7 IHC on serial sections from human TMAs. u.d., undetected. Scale bars: 10 μm. (**B**) Quantification of *FBW7* mRNA on CRC TMAs in 28 tissue cores positive for a control *PPIB* mRNA. (**C**) Quantification of FBW7 IHC in tissue cores from **A**. (**D**–**F**) Endogenous FBW7 interacts with endogenous USP9X and USP9X interacts with endogenous FBW7 in HEK293 cells. Black line in **D** indicates noncontiguous lanes from the same gel. siFBW7 control in **F** confirms antibody specificity of FBW7 and represents a negative control for FBW7-USP9X interaction. (**G**) Endogenous USP9X interacts with epitope-tagged FBW7 isoforms α and β.

To investigate the prevalence of altered posttranscriptional regulation of FBW7 protein in human CRC, we performed in situ hybridization (RNAscope) and IHC for FBW7 on serial sections from tissue microarrays (TMAs) of patients with CRC. A control RNA probe, for *PPIB* (cyclophilin B), was clearly detected in all examined cases, confirming the presence of intact mRNA on individual TMA cores (Figure 1A). In contrast, *FBW7* mRNA levels differed greatly among samples. Whereas 50% of cases showed high *FBW7* mRNA signal, in the other 50% *FBW7* mRNA was present at low levels or was undetectable (Figure 1, A and B).

The majority of *FBW7-*low mRNA cases (with one exception) had undetectable or weak FBW7 immunostaining (Figure 1A, case 1), as expected. Intriguingly, most samples (11 of 14, 78.5 %) with high *FBW7* mRNA levels had weak to undetectable FBW7 immunostaining, while only a few (3 of 14, 21.5%) had strong immunostaining (Figure 1A, cases 2 and 3, and Figure 1C). Thus, altered transcriptional as well as posttranscriptional mechanisms resulted in restricted FBW7 levels in most (24 of 28, 86%) of the human CRCs analyzed.

### Identification of USP9X as an FBW7 binding partner.

To understand the molecular mechanism underlying FBW7 regulation at the protein level, we decided to examine the FBW7 interactome in human cells. For this, we immunoprecipitated (IP) endogenous FBW7 (using an FBW7α-specific antibody) from HEK293 cells and analyzed the eluted samples by mass spectrometry (MS). In addition to the bait, FBW7, the proteins identified by MS analysis that were most enriched in the FBW7 IP (over IP with control IgG) included components of the SCF-type E3 ubiquitin ligase complex, including SKP1, and several known SCF(FBW7) substrates, including c-JUN, mTOR, and DEK (Supplemental Figure 1A and Supplemental Table 1; supplemental material available online with this article; https://doi.org/10.1172/JCI97325DS1). We also identified the DUB USP9X enriched in the FBW7 IP eluates. Based on its tissue-specific roles in both tumor promotion (32) and suppression (33), we focused on USP9X for further validation and biochemical evaluation. First, we confirmed the interaction between endogenous FBW7 and USP9X by immunoprecipitation of FBW7, followed by Western blot analysis (Figure 1, D–F). Conversely, USP9X overexpressed in HEK293 cells pulled down endogenous FBW7 (Figure 1E). Second, to check whether USP9X interacted with other isoforms of FBW7, we overexpressed Flag-tagged FBW7α, -β, and -γ, respectively, in HEK293 cells and confirmed USP9X interaction with α and β isoforms but not with FBW7γ (Figure 1G).

### USP9X counteracts FBW7 ubiquitylation.

**Figure 2:**
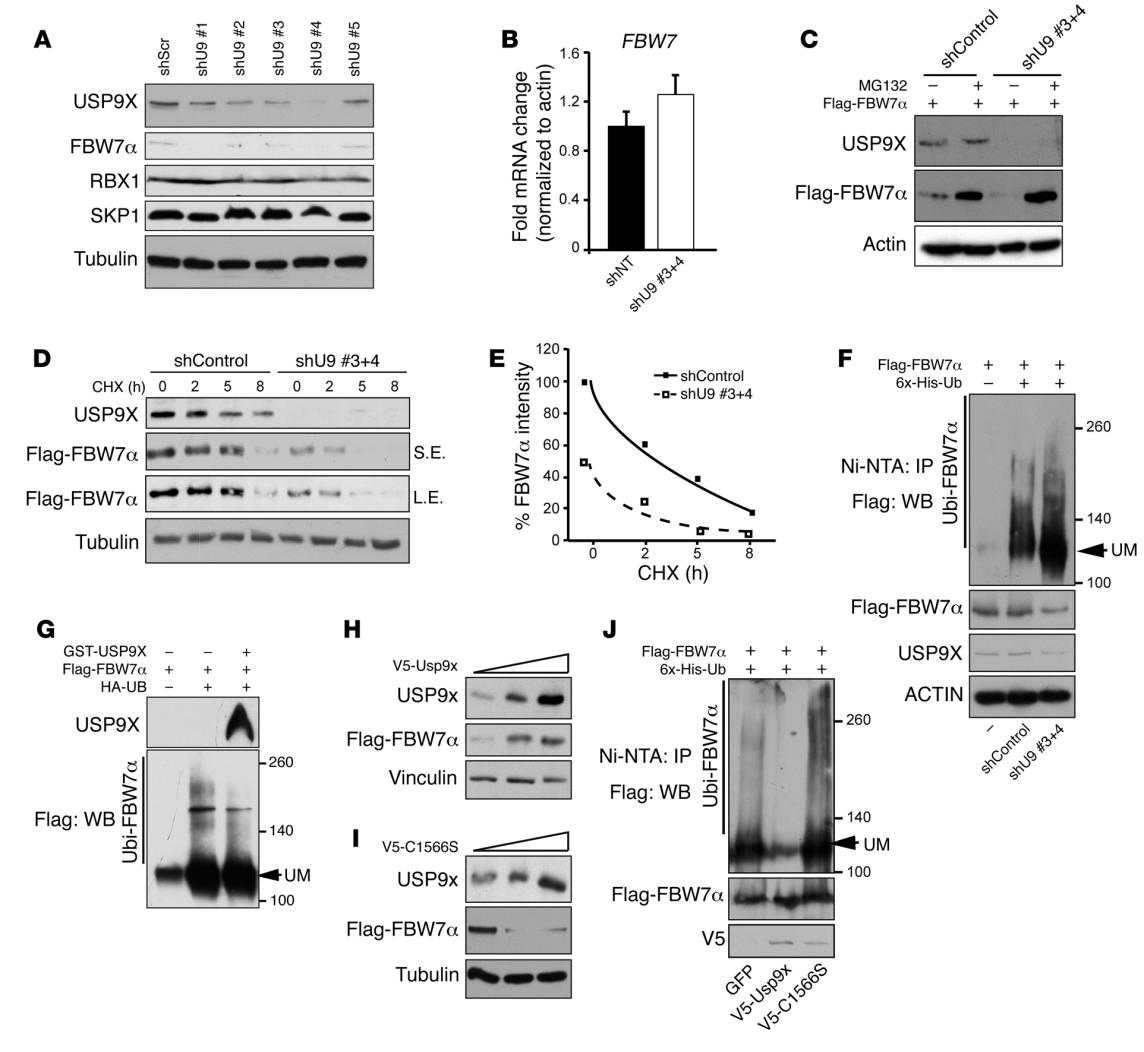
FBW7 is a USP9X substrate. (**A** and **B**) USP9X knockdown using multiple shRNAs reduced FBW7 protein levels, with no effect on its mRNA. shScr, scrambled control; shNT, nontargeting control. (**C**) MG132 rescues reduced FBW7 protein levels in USP9X-silenced cells. (**D**) USP9X knockdown reduced the half-life of FBW7 protein. CHX, cycloheximide. (**E**) Quantification of 3 independent experiments performed as in **D**. (**F**) Increased ubiquitylation (Ubi) of FBW7 in *USP9X*-silenced cells. UM, unmodified. (**G**) In vitro deubiquitylation of FBW7 by recombinant GST-USP9X. (**H** and **I**) Western blots for Flag-FBW7α in HEK293 cells co-overexpressing WT (V5-Usp9x) or catalytically dead (V5-C1566S) mouse Usp9x. (**J**) Western blots on Ni-NTA pulldown samples from cells cotransfected with Flag-FBW7α and the indicated constructs. All experiments were done in HEK293 cells.

Next, we investigated the biochemical significance of the FBW7-USP9X interaction. If USP9X were an FBW7 substrate, shRNA-mediated knockdown of *FBW7* should result in USP9X stabilization. However, shRNA-mediated knockdown of *FBW7* had no effect on USP9X levels (Supplemental Figure 1B). Conversely, multiple different shRNAs targeting *USP9X* caused a sharp decrease in endogenous FBW7, whereas RBX1 and SKP1 protein and *FBW7* mRNA levels were unaffected (Figure 2, A and B). These results suggest that USP9X may control FBW7 protein stability and that FBW7 could be a USP9X substrate.

If USP9X-FBW7 interaction were to regulate FBW7 protein stability by reducing its proteasomal degradation, then a proteasome inhibitor (MG132) should restore the levels of FBW7 in *USP9X*-silenced cells. Indeed, levels of Flag-FBW7α and Flag-FBW7β were restored by MG132 in *USP9X-*silenced cells (Figure 2C and Supplemental Figure 1C). In addition, *USP9X* depletion resulted in a striking reduction in Flag-FBW7α protein stability, as judged by a cycloheximide time course experiment (Figure 2, D and E).

To further understand FBW7 regulation by USP9X, we performed Ni-NTA pulldowns on lysates from HEK293 cells cotransfected with 6x-His–ubiquitin, Flag-FBW7α, and a scrambled (control) shRNA or a combination of 2 shRNAs targeting *USP9X* (shU9 #3+4). Knockdown of *USP9X* increased Flag-FBW7α ubiquitylation (Figure 2F). Conversely, addition of recombinant GST-USP9X to eluted HA-ubiquitylated Flag-FBW7α reduced FBW7 ubiquitylation in vitro but had only a negligible effect on eluted HA-ubiquitylated Flag–c-JUN, confirming substrate specificity of USP9X for FBW7 (Figure 2G and Supplemental Figure 2A).

FBW7 protein levels are regulated by proteasomal degradation (34), suggesting that FBW7α is polyubiquitylated via Lys48 (K48) linkage. To formally demonstrate this, we performed a ubiquitin chain restriction analysis (Ubi-CRest) using purified polyubiquitylated Flag-FBW7α as a substrate for K48- and K63-specific DUBs (OTUB1 and AMSH, respectively) and USP9X. The high-molecular-weight species appearing in Flag-FBW7 Western blots were indeed polyubiquitylated FBW7α, since a promiscuous DUB, USP2, cleaved all the conjugated ubiquitins off FBW7 (Supplemental Figure 2, B and C). In addition, only OTUB1 and USP9X, but not AMSH, cleaved conjugated ubiquitins off FBW7α, suggesting that the majority of polyubiquitylated FBW7α is linked via K48. This was confirmed using a K48 linkage-specific antibody (Supplemental Figure 2C).

Flag-FBW7α was stabilized by WT mUsp9x (V5-Usp9x) (Figure 2H) but not by a V5-C1566S catalytically dead mutant (Figure 2I). Additionally, mUsp9x expression greatly reduced ubiquitylation of Flag-FBW7α, whereas the catalytically dead mutant V5-C1566S slightly increased FBW7 ubiquitylation, perhaps indicating a dominant negative effect (Figure 2J). Thus, USP9X DUB activity antagonizes degradative K48-linked FBW7 polyubiquitylation.

### USP9X negatively regulates SCF(FBW7) substrates.

**Figure 3:**
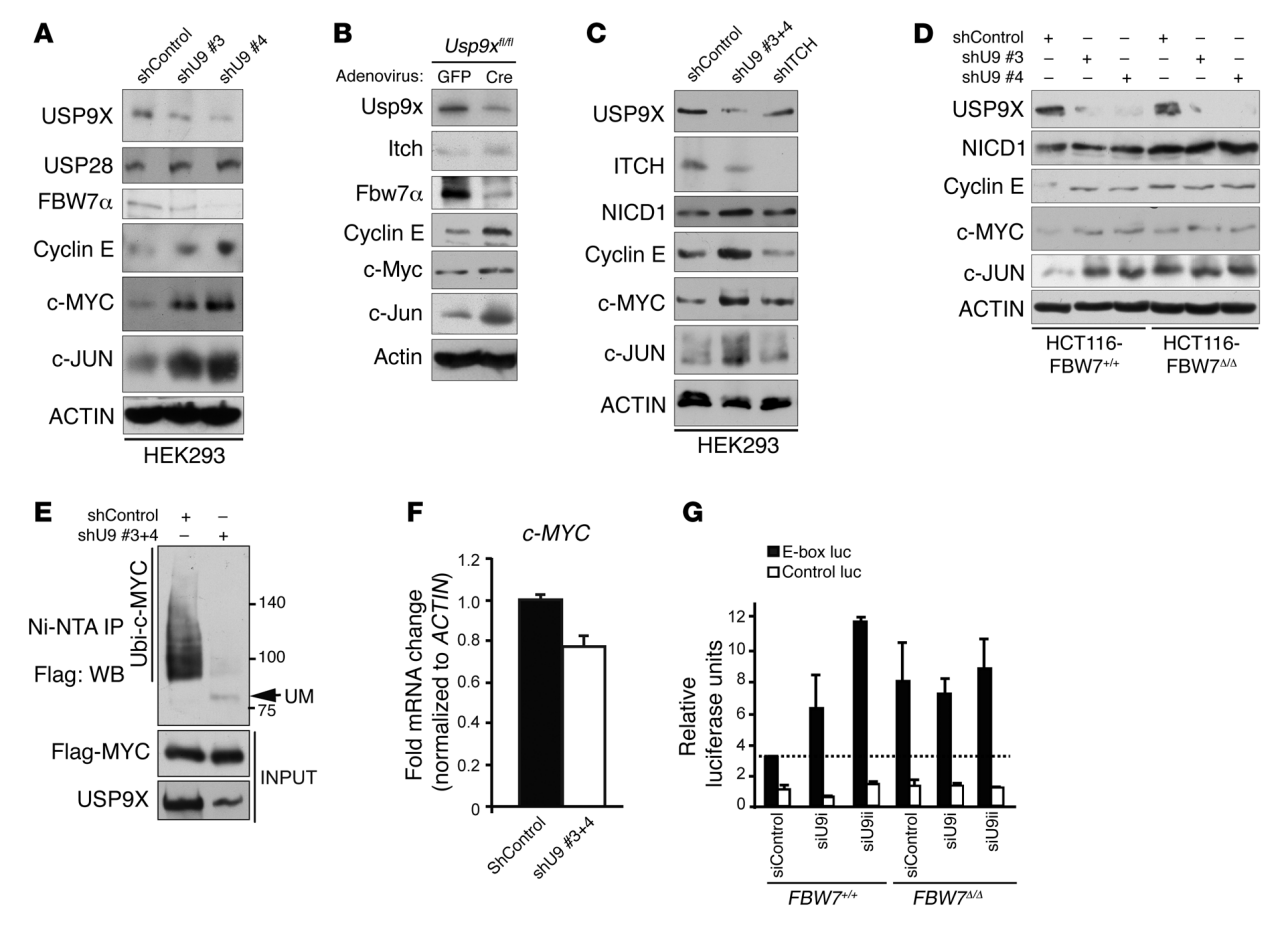
USP9X negatively regulates SCF(FBW7) substrates. (**A**) Accumulation of SCF(FBW7) substrates in *USP9X*-silenced HEK293 cells. (**B**) Accumulation of SCF(Fbw7) substrates in *Usp9x*-knockout murine adult fibroblasts. (**C**) Western blots showing levels of SCF(FBW7) substrates in *USP9X*- and *ITCH*-silenced HEK293 cells. (**D**) Accumulation of SCF(FBW7) substrates with *USP9X* silencing was abolished in the HCT116-*FBW7^Δ/Δ^* CRC cell line. (**E** and **F**) Western blots for ubiquitylated c-Myc on Ni-NTA pulldown from HEK293 cells overexpressing Flag–c-Myc and 6x-His–tagged ubiquitin and cotransfected with the indicated shRNAs. *c-MYC* mRNA levels in the same cells are shown in **F**. (**G**) c-MYC luciferase activity in HCT116 cells with the indicated genotypes and siRNA treatments. Mean of 2 independent experiments is shown.

Next, we examined the effect of *USP9X* loss on SCF(FBW7) substrates. Knockdown of *USP9X* using 2 different shRNAs caused a sharp decrease in endogenous FBW7 and concomitantly stabilized the SCF(FBW7) substrates c-MYC, c-JUN, and cyclin E in 293T and HCT116 cells (Figure 3A and Supplemental Figure 3). Protein levels of USP28, another DUB previously reported to differentially regulate FBW7 stability (35), were unaffected by USP9X knockdown (Figure 3A). To investigate USP9X function in primary cells, we isolated mouse adult fibroblasts (MAFs) from *Usp9x^fl/fl^* mice and treated them with either adeno-*GFP* (control) or adeno-*Cre* (*Cre*) to induce recombination. In vitro deletion of *Usp9x* in MAFs resulted in a decrease in the level of Fbw7 protein and accumulation of SCF(Fbw7) substrates (Figure 3B). Protein levels of Itch, an E3 ligase previously shown to be a USP9X substrate (36), were unaffected in these cells. In addition, shRNA-mediated *ITCH* knockdown, unlike *USP9X* knockdown, did not result in stabilization of SCF(FBW7) substrates (Figure 3C). To confirm the relevance of FBW7 in the regulation of SCF(FBW7) substrates by USP9X, we knocked down *USP9X* using 2 different shRNAs in HCT116-*FBW7^+/+^* and HCT116-*FBW7^Δ/Δ^* cells. Whereas *USP9X* knockdown increased NICD1, cyclin E, c-MYC, and c-JUN protein levels in HCT116-*FBW7^+/+^* cells, in HCT116*-FBW7^Δ/Δ^* cells the protein levels of these substrates were already increased and were not further affected by *USP9X* knockdown (Figure 3D). These results suggest that USP9X controls the protein stability of SCF(FBW7) substrates by direct regulation of FBW7 protein.

Consistent with the notion that USP9X negatively regulates c-MYC protein stability, c-MYC ubiquitylation was reduced in *USP9X*-knockdown cells, with no effect on its mRNA (Figure 3, E and F). In agreement with these findings, USP9X silencing using 2 different siRNAs increased c-MYC transcriptional activity in HCT116-*FBW7^+/+^* but not in HCT116-*FBW7^Δ/Δ^* cells, as indicated by a dual luciferase c-MYC reporter assay (Figure 3G). Thus, USP9X negatively regulates c-MYC activity via direct stabilization of FBW7.

### Usp9x controls tissue homeostasis in murine intestine.

**Figure 4:**
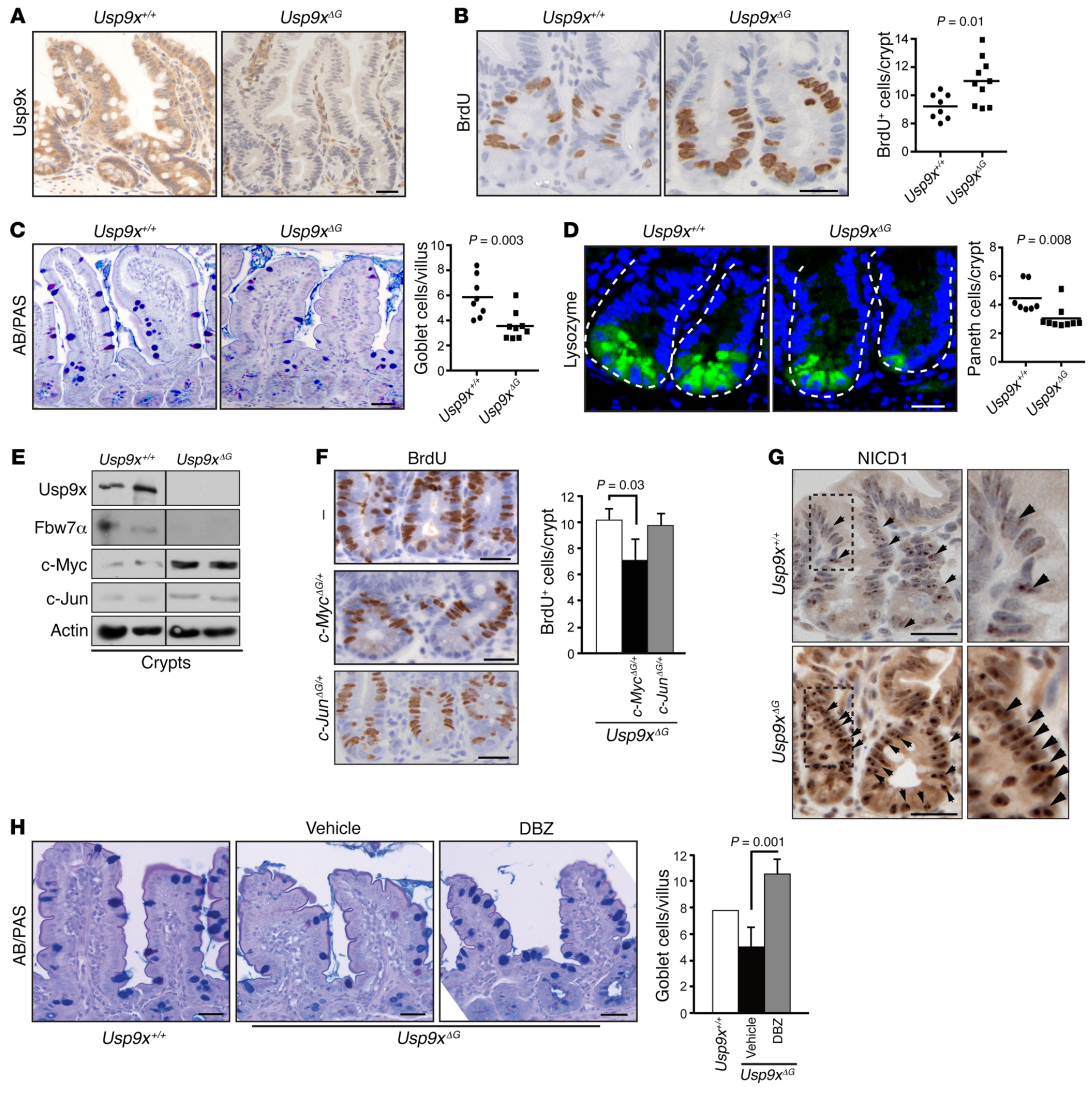
Usp9x controls intestinal tissue homeostasis. (**A**–**D**) Representative IHC sections for Usp9x (**A**), transit-amplifying cells (BrdU, **B**), goblet cells (AB/PAS, **C**), and Paneth cells (lysozyme, **D**) in WT (*Usp9x^+/+^*) and *Usp9x*-knockout mouse gut. Scale bars: 50 μm. Right panels, quantification from **B**–**D**; *n* = 5–8 mice/group. Dashed lines outline crypts. (**E**) Western blots for the indicated proteins in freshly isolated crypts from WT and *Usp9x*-knockout mouse gut. Black line indicates noncontiguous lanes from the same gel. (**F**) BrdU staining on gut cross sections from *Usp9x^ΔG^* mice. Scale bars: 50 μm. Right: quantification from **F**; *n* = 3–4 mice/group. (**G**) IHC for NICD1 in mouse gut. Scale bars: 50 μm. Right panels are ×2 magnification of the dashed areas on the left. (**H**) AB/PAS staining on guts from the indicated mice treated with a vehicle or a Notch inhibitor (DBZ). Scale bars: 50 μm. Right: quantification from **H**; *n* = 3–4 mice/group. Data are shown as mean + SD, and statistical significance was calculated by Student’s *t* test.

To explore Usp9x-mediated Fbw7 regulation in vivo, we crossed *Usp9x^fl/fl^* mice with the gut-specific *Villin*-*Cre* mouse and analyzed transverse sections of gut from the resulting *Usp9x^fl/fl^* *Villin*-*Cre* and *Usp9x^fl/y^* *Villin*-*Cre* mice (*Usp9x^ΔG^* mice). IHC analyses revealed uniform expression of Usp9x throughout the crypts and villi in the gut of the control mice, whereas no staining was detected in the intestinal epithelium of *Usp9x^ΔG^* mice (Figure 4A). Cellular analysis revealed a significant increase in proliferating cells in *Usp9x^ΔG^* small intestine crypts, evident from IHC following a BrdU pulse (Figure 4B) and from the increased numbers of MCM6^+^ cells extending from the crypt-villus junction down to the crypt base (Supplemental Figure 4A). Similar results were obtained in colonic crypts from *Usp9x^ΔG^* mice (Supplemental Figure 4B). Goblet and Paneth cell numbers in *Usp9x^ΔG^* villi and crypts were reduced (Figure 4, C and D), as observed in *Fbw7*-deficient murine intestine (28). However, numbers of crypt base columnar cells, enteroendocrine cells, and enterocytes were similar in WT and *Usp9x^ΔG^* mice as judged by in situ hybridization of *Olfm4*, and by chromogranin and alkaline phosphatase staining (Supplemental Figure 4, C–E). There was a striking reduction in Fbw7 protein, while *Fbw7* mRNA was unaffected (Figure 4E and Supplemental Figure 4F), and this was accompanied by c-Myc and c-Jun accumulation in *Usp9x^ΔG^* crypts (Figure 4E).

To establish which SCF(Fbw7) substrates mediate hyperproliferation of TA cells and reduced secretory cell differentiation in *Usp9x^ΔG^* crypts, we crossed either *c-Myc^fl/fl^* or *c-Jun^fl/fl^* mice with *Usp9x^ΔG^* mice and compared the proliferation and differentiation in the gut of the resulting *c-Myc*^ΔG/+^ *Usp9x^ΔG^* and *c-Jun^ΔG/+^* *Usp9x^ΔG^* mice with those of *Usp9x^ΔG^* mice. Deletion of one *c-Myc*, but not one *c-Jun* allele, was sufficient to reduce TA cell numbers to WT levels, whereas neither *c-Myc* nor *c-Jun* heterozygosity had any effect on the goblet cell numbers in *Usp9x^ΔG^* intestine (Figure 4F and Supplemental Figure 5A). Instead, reduced differentiation of TA cells in *Usp9x^ΔG^* intestine was most likely a consequence of increased Notch activity (Figure 4G), since treating *Usp9x^ΔG^* mice with the γ-secretase inhibitor dibenzazepine (DBZ) restored the number of goblet cells even beyond WT levels, with no effect on TA cell proliferation (Figure 4H and Supplemental Figure 5B). Hence, Usp9x controls tissue homeostasis in the murine gut by negatively regulating c-Myc and Notch1, most likely via stabilization of Fbw7.

### Usp9x is required for tissue regeneration after acute colitis.

**Figure 5:**
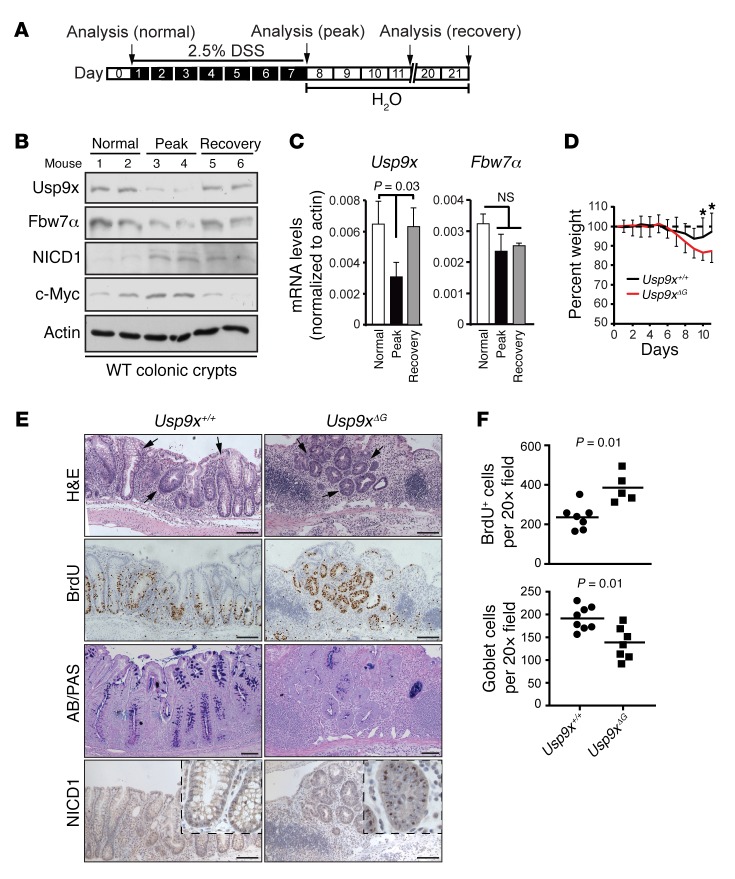
Usp9x is required for tissue regeneration during acute colitis. (**A**) Schematic of acute colitis protocol. (**B**) Western blots for the indicated proteins in 6 independent WT mice fed with (day 7, “peak” and day 21, “recovery”) or without (day 0, “normal”) 2.5% DSS in water. (**C**) qRT-PCR analysis for *Usp9x* and *Fbw7* mRNA in different phases of colitis from the experiment in **B**. Statistical significance calculated by 1-way ANOVA. (**D**) Weight curves from DSS-induced colitis experiment in the indicated mice; *n* = 7–8/group. *P < 0.05. (**E**) Representative IHC sections for H&E, BrdU, AB/PAS, and NICD1 from *Usp9x^+/+^* and *Usp9x^ΔG^* mice. Scale bars: 100 μm. (**F**) Quantification of BrdU^+^ and AB/PAS^+^ cells from the experiment in **D**. Data are presented as mean; statistical significance was calculated by Student’s *t* test in **D** and **F**.

To further explore the role of Usp9x in tissue maintenance, damage, and repair, we used a standard model of dextran sodium sulfate–induced (DSS-induced) acute colitis in mice (Figure 5A). We first tested the protein and mRNA levels of *Usp9x* and *Fbw7* in different disease phases of DSS-induced colitis. For this, we fed WT littermates 2.5% DSS in drinking water for 7 days and analyzed tissues by Western blot and qRT-PCR at 0 (normal), 7 (disease peak), and 21 (recovery) days. Notably, Usp9x and Fbw7 protein levels were dramatically reduced during the peak phase of colitis (day 7) and were restored to normal levels at later stages (Figure 5B). Conversely c-Myc and NICD1 accumulated during the disease peak and were reduced when the tissue recovered (Figure 5B). Strikingly, *Usp9x* mRNA was reduced by around 50% during the disease peak, whereas *Fbw7* mRNA was not significantly affected throughout the experiment (Figure 5C), suggesting that decreased Fbw7 protein levels in response to colitis are the consequence of reduced *Usp9x* expression.

After onset of DSS-induced colitis, *Usp9x^ΔG^* mice showed reduced body weight (Figure 5D), suggesting impaired intestinal regeneration. IHC revealed highly proliferative, disorganized crypt structures in colon ulcers from those mice and a substantial increase in BrdU^+^ proliferating cells (Figure 5, E and F). In contrast, the number of goblet cells produced after regeneration in the *Usp9x^ΔG^* intestine was low, most likely due to increased NICD1 protein levels (Figure 5, E and F). Thus, Usp9x may control the response to injury in murine colon by orchestrating the protein levels of SCF(Fbw7) substrates to ensure the transition from regenerating progenitor to cell differentiation.

### Usp9x suppresses CRC.

**Figure 6:**
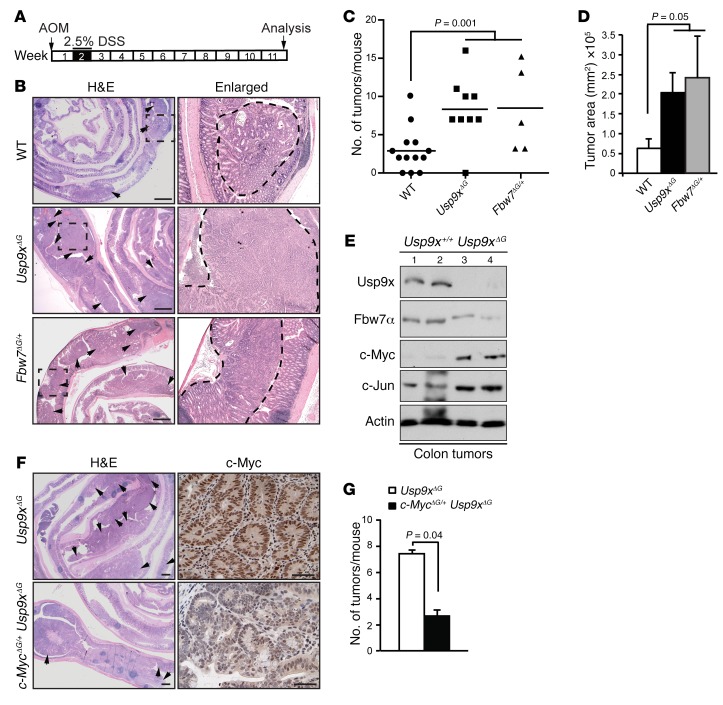
Usp9x suppresses CRC. (**A**) Schematic of AOM/DSS-induced CRC model. (**B**) H&E on sections from transformed colons of the indicated genotypes. Arrowheads indicate tumors. Scale bars: 100 mm. Right panels: ×4 magnification of the dashed areas on the left; dashed lines outline tumors. (**C**) Number of tumors in each mouse from experiment in **B**; *n* = 5–13/group. (**D**) Tumor area represented as mm^2^ in mice from **B**; *n* = 5–13/group. (**E**) Western blots showing levels of indicated proteins in 4 independent tumors from **B**. (**F**) H&E and c-Myc staining on sections from transformed colons of the indicated genotypes. Scale bars: 50 mm (H&E); 100 μm (c-Myc). Arrowheads indicate tumors. (**G**) Average number of tumors in indicated mice from experiment in **F**; *n* = 3–5/group. Data are presented as mean; statistical significance calculated by 1-way ANOVA in **C** and **D**, and by Student’s *t* test in **G**.

The regulation of Fbw7 by Usp9x and increased proliferation of colonic crypts after acute colitis in *Usp9x^ΔG^* mice prompted us to test the role of Usp9x in colitis-mediated CRC. In the azoxymethane (AOM)/DSS-induced CRC model (Figure 6A), *Usp9x^ΔG^* mice showed an almost 3-fold increase in tumor burden, including increases in tumor number and tumor area compared with WT controls (Figure 6, B–D). Similar results were obtained with *Fbw7^ΔG/+^* mice (Figure 6, B–D). Both the *Usp9x^ΔG^* and *Fbw7^ΔG/+^* mice showed reduced body weight throughout the experimental period, suggestive of a more advanced disease (Supplemental Figure 6, A and B). Fbw7 protein levels were reduced in *Usp9x^ΔG^* tumors, with a concomitant increase in c-Myc and c-Jun, as judged by Western blot analysis (Figure 6E). To validate the role of c-Myc in AOM/DSS-mediated tumorigenesis in *Usp9x*^ΔG^ mice, we inactivated one *c-Myc* allele in *Usp9x^ΔG^* mice and compared the number of tumors in *c-Myc^ΔG/+^* *Usp9x*^ΔG^ mice with those in *Usp9x^ΔG^* mice. Indeed, *c-Myc* heterozygosity was sufficient to reduce the tumor burden to WT levels in *Usp9x*^ΔG^ mice, with a clear reduction of c-Myc staining in *c-Myc^ΔG/+^* *Usp9x^ΔG^* tumors (Figure 6, F and G). Thus, negative regulation of c-Myc by Usp9x via direct stabilization of Fbw7 protects mice from colitis-mediated CRC.

### Reduced USP9X strongly correlates with reduced FBW7 protein and poor prognosis in human cancers.

**Figure 7:**
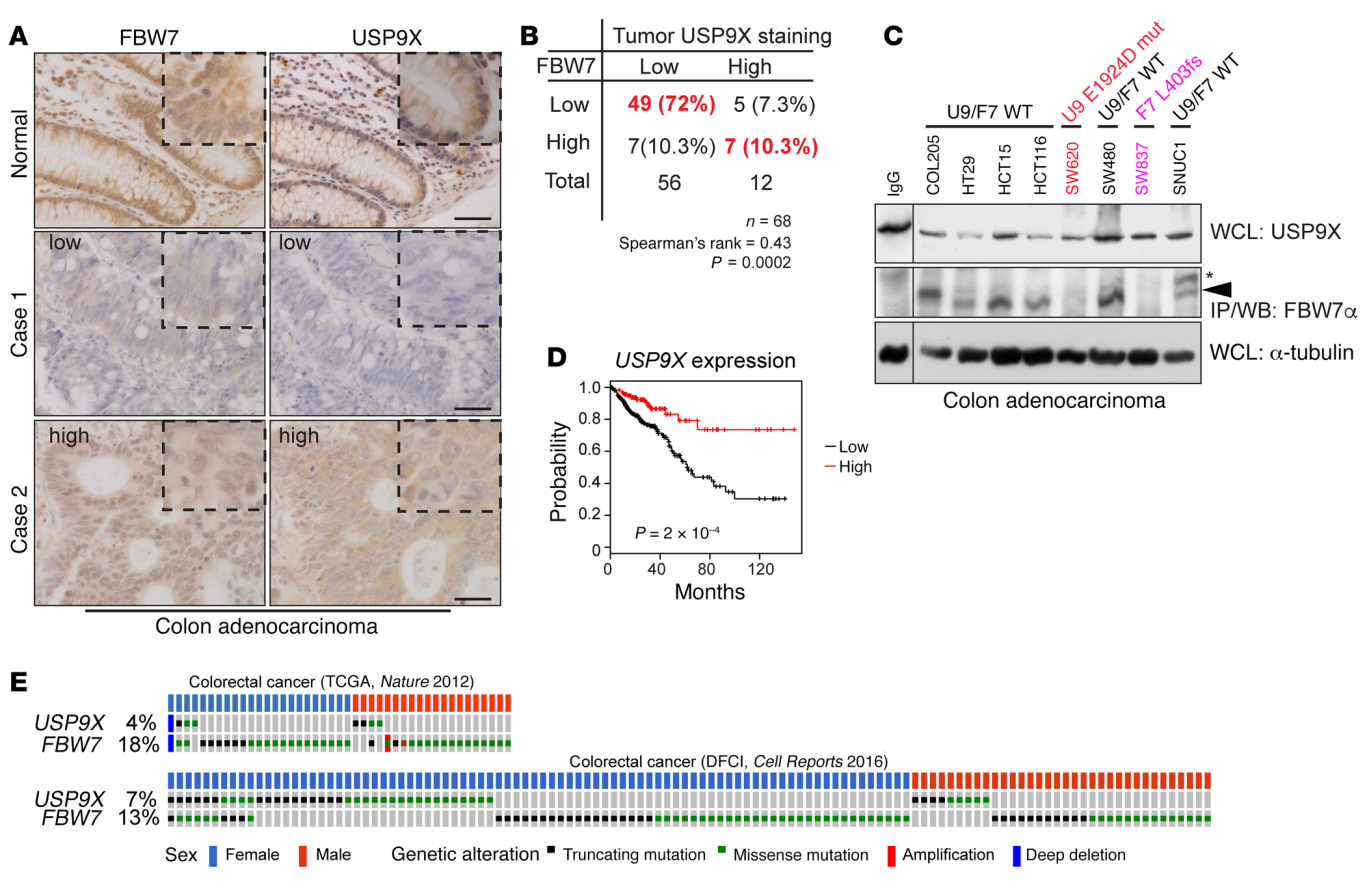
Reduced USP9X is associated with poor prognosis in human CRC. (**A**) IHC for the indicated proteins on human CRC TMAs, including associated normal tissue. Scale bars: 50 μm. Insets show x2 magnifications of areas in the main image. (**B**) Quantification of staining intensities from sections in **A**, and their Spearman’s rank correlation. (**C**) Western blots showing positive correlation of USP9X and FBW7 protein levels in 8 different CRC cell lines. Red: *USP9X* mutant, pink: *FBW7* truncation mutant (homozygous) cell lines. Black line indicates noncontiguous lanes from the same gel. (**D**) Kaplan-Meier plot showing comparison of survival between *USP9X*-low and -high expression groups of CRC patients. Data are from TCGA. (**E**) Mutational status of *USP9X* and *FBW7* in CRC patients. Data are from the TCGA dataset of 212 patients and the DFCI Colorectal Adenocarcinoma dataset of 619 patients (published in refs. 6, 45 [*Nature* 2012 and *Cell Reports* 2016, respectively]). Data viewed with cBioPortal. Only samples with alterations in either *USP9X* or *FBW7* are shown. Fisher’s exact test *P* = 0.01 for females and *P* = 1 for males. WCL, whole cell lysate.

To test the relationship between USP9X and FBW7 in human cancers, we stained serial sections of human colon cancer TMAs for USP9X and FBW7 (Figure 7A). Both USP9X and FBW7 were readily detected in adjacent normal tissue in all cases. In CRC tumor tissue, USP9X and FBW7 showed a strong correlation in staining intensity, with 72% showing concomitant downregulation (Figure 7A, case 1, and Figure 7B). In a smaller percentage of cases, both FBW7 and USP9X were strongly expressed (Figure 7A, case 2, and Figure 7B). The correlation between FBW7 and USP9X protein expression was also evident in a panel of 8 human CRC cancer cell lines. FBW7 was only detectable by Western blot in cell lines with higher USP9X protein (Figure 7C). Interestingly, this correlation was lost in 2 cell lines harboring a *USP9X* or *FBW7* mutation, respectively, further confirming the positive regulation of FBW7 by USP9X (Figure 7C).

In support of its protumorigenic effect in mice, we found that low *USP9X* expression was strongly associated with poor survival in human CRC (Figure 7D). Moreover, *USP9X* was mutated in a small percentage of CRC patients. Interestingly, concurrent *FBW7* mutations predominantly occurred in females (Fisher’s exact test, *P* = 0.01) and not in males (*P* = 1) (Figure 7E), suggesting that incomplete *USP9X* inactivation caused by random X inactivation in females may favor a second genetic mutation in the *FBW7* gene to fully inactivate the function of the USP9X-FBW7 axis. Taken together, these data support the notion that USP9X acts as a tumor suppressor in the intestine via positive regulation of FBW7.

## Discussion

*FBW7* is a haploinsufficient tumor suppressor gene (37) mutated in a wide variety of human cancers, but mechanisms regulating FBW7 protein stability and activity are largely unknown. In this study, we identify a mechanism of FBW7 protein regulation by the DUB USP9X. USP9X antagonized FBW7 ubiquitylation and protected mice from CRC. Importantly, reduced USP9X expression predicted poor survival in human cancers. Thus, USP9X functions indirectly as a tumor suppressor, by controlling the protein stability of FBW7.

USP9X is a C19-peptidase family protein with known DUB activity (36). It cleaves both K48- and K63-mediated linkages. USP9X can have both pro- and antitumorigenic functions depending on tumor type. For example, it functions as an oncogene in multiple myeloma and lymphoma (32); however, it is a potent tumor suppressor in the pancreas (33). Our data demonstrate that in addition to the pancreas, USP9X is also a tumor suppressor in the intestine. Although the E3 ligase Itch was shown to mediate the antitumor effects of USP9X in the pancreas (33), our data demonstrate that USP9X can prevent intestinal cancer by directly regulating the stability of FBW7 protein.

Our data indicate that regulation of Fbw7 plays a major role in mediating the loss-of-Usp9x phenotype in the intestine. Indeed, Fbw7 protein levels were dramatically low in isolated crypts from *Usp9x^ΔG^* mice (Figure 4E), and the gut phenotype of *Usp9x^ΔG^* mice, characterized by reduced differentiation and increased proliferation, resembles that of *Fbw7^ΔG^* mice (28). As in *Fbw7^ΔG^* intestine, c-Myc and Notch1 appear to be the main mediators of the *Usp9x^ΔG^* phenotypes.

The positive regulation of FBW7 by USP9X reinforces the importance of protein turnover and regulation in tissue homeostasis and cancer. The deubiquitylation of FBW7 protein by the DUB activity of USP9X is a direct and rapid mechanism of transiently regulating FBW7 protein levels and thus its activity. Additionally, this mechanism may allow control of FBW7 stability in an isoform-specific manner, as USP9X interacts with FBW7α and -β, but not with FBW7γ (Figure 1G).

The regulation of Fbw7 by Usp9x also appears to be crucial during intestinal regeneration. Indeed, *Usp9x* was transcriptionally downregulated during the peak phase of colitis, and this resulted in a reduction in FBW7 protein levels with a concomitant increase in c-Myc and NICD1 (Figure 5, B and C). Importantly, *Usp9x* mRNA levels were restored after recovery from colitis, and c-Myc and NICD1 returned to normal. As expected, *Usp9x^ΔG^* colonic ulcers after acute colitis were marked by increased proliferation and decreased secretory differentiation, most likely because the normalization of c-Myc and NICD1 protein is delayed in the absence of Usp9x, and the damaged intestine was locked in a proliferative state (Figure 5E). Thus, Usp9x may function as an “emergency switch” in the injured intestine, allowing high proliferation required during regeneration and subsequently mediating the transition to differentiation by restoring the levels of Fbw7.

Importantly, the regulation of FBW7 by USP9X has implications in human intestinal cancer. We found that a substantial number of human CRC tumors expressed normal levels of *FBW7* mRNA, but FBW7 protein was either undetected or weakly expressed (Figure 1A). In these patients FBW7 function appears to be compromised regardless of *FBW7* mutations. Our data suggest that USP9X is an important mediator of FBW7 protein stability. Of 56 patients with low FBW7 IHC staining, 49 had low USP9X protein levels (Figure 7B). The strong and direct correlation between USP9X and FBW7 in human CRC, together with our biochemical characterization of FBW7 as a USP9X substrate, strongly suggests a causal role for USP9X loss in intestinal tumorigenesis. Consistent with this idea, we found that low *USP9X* expression was strongly associated with poor survival in colon cancer (Figure 7D).

The incidence of CRC in males is higher than in females (38). One plausible explanation for this apparent sex bias is the presence of tumor suppressor genes on the X chromosome that protect females from the effects of loss-of-function mutations (39). In agreement with this idea, we find that a significant percentage of female, but not male, colon cancer patients with *USP9X* mutations carry a concomitant *FBW7* mutation (Figure 7E). Because males have only one *USP9X* allele, this supports the notion that females may require an additional “hit” in the *FBW7* gene to achieve a sufficient decrease in function of the USP9X-FBW7 axis. Thus, USP9X is a crucial regulator of FBW7 protein levels and function, and the degradation of FBW7 in the absence of USP9X is a mechanism of tumor promotion.

## Methods

### RNA in situ hybridization for FBW7.

RNAscope was performed on human CRC TMAs (CO811, US Biomax) as recommended by the manufacturer (ACD; https://acdbio.com, user manual doc. 322310-QKG. Briefly, target retrieval was performed for 15 minutes, followed by RNAscope Protease Plus incubation for 30 minutes on the FFPE Sample Preparation. The counterstaining and mounting of the slides was performed on a Tissue-Tek Prisma staining machine.

### MS.

Subconfluent HEK293 cells were treated with a proteasome inhibitor (MG132) for 6 hours. Cells were washed with ice-cold PBS and lysed in 1× Cell Lysis Buffer (CLB; Cell Signaling Technology, catalog 9803) supplemented with protease inhibitors, PMSF (1 mM), and sodium fluoride (5 mM). FBW7α was immunoprecipitated with 5 μg/ml FBW7α antibody (Bethyl Laboratories Inc., A301-720A). Rabbit IgG at 5 μg/ml was used as a negative control. Immunoprecipitated complexes were washed 5 times in 1× ice-cold CLB. Eluted samples were run on 12 % Tris-HCl gel for 5–10 minutes at 150 volts. Gels were washed with distilled water, fixed, Coomassie stained, and recovered in distilled water. Sample bands were diced with a sterile razor blade on a glass slide prewashed with 100% methanol and stored in distilled water for MS analyses, as previously described (40).

### Source of cell lines.

HEK293, HCT116-*FBW7^+/+^* and HCT116-*FBW7^Δ/Δ^*, COL205, HT29, HCT115, SW620, SW480, SW837, and SNUC1 cell lines were obtained from Cell Services at the Francis Crick Institute.

### Western blot analysis and qRT-PCR.

Immunoblots were carried out as previously described (28). Antibodies against USP9X (Bethyl Laboratories Inc., catalog A301-351A), FBW7α (Bethyl Laboratories Inc., A301-720), c-Jun (BD Biosciences, 610326), active Notch-1 (Abcam, ab8925), c-Myc and cyclin E (Santa Cruz Biotechnology Inc., sc-788 and sc-481), α-tubulin (Abcam, ab7291), Apu2 clone, K48-linkage (MilliporeSigma, 05-1307), and β-actin (MilliporeSigma, A3854) were used.

For qRT-PCR analysis, total mRNA was isolated from dissected ileum as previously described (28). Results normalized to β-actin were presented as fold induction relative to control. The primers used for qRT-PCR analysis were previously published (24, 28, 33).

### Ubiquitylation assays.

In HEK293 cells, 6x-His–ubiquitin was overexpressed along with Flag-FBW7 and a control or *USP9X*-specific shRNA (shU9 #3+4). Forty-eight hours after transfection, cells were treated with MG132 for 6 hours, washed, collected, and lysed in buffer A (6M guanidine hydrochloride, 0.1 M Na_2_HPO_4_, and 0.1 M NaH_2_PO_4_) supplemented with 20 mM imidazole. The ubiquitylated proteins were precipitated using Ni-NTA beads (QIAGEN) for 2 hours at room temperature, washed 3 times in buffer A, twice in buffer A/TI (1 volume buffer A + 3 volume 25 mM Tris-HCl + 10 mM imidazole), and twice in buffer TI (25 nM Tris-HCl + 10 mM imidazole). The bead-bound ubiquitylated proteins were eluted in 1M imidazole-containing sample buffer, resolved on 7.5% Tris-HCl gel, and transferred to nitrocellulose membranes. The blots were probed with Flag-HRP antibody (MilliporeSigma, catalog A8592).

For in vitro deubiquitylation, Flag-FBW7 was co-overexpressed with HA-ubiquitin for 48 hours in HEK293 cells. The transfected cells were treated with MG132 as above and the ubiquitylated proteins were immunoprecipitated using anti-HA affinity agarose (MilliporeSigma, catalog A7470) overnight. The bead-bound HA-ubiquitylated complexes were washed once in 1× CLB and incubated with 0.5 mg/ml HA-peptide (MilliporeSigma, I2149) for 1 hour at 4°C with constant shaking. Flag-FBW7 was immunoprecipitated from 2 sequentially pooled HA-peptide elutions using Flag-M2 affinity beads (MilliporeSigma, F2426) for up to 4 hours at 4°C. The polyubiquitinated Flag-FBW7–bound M2 beads were then washed 3 times in PBS-Tween (0.05%) and eluted twice with 0.25 mg/ml Flag peptide (MilliporeSigma, F4799) in TNT-300 buffer (50 mM Tris pH 7.4, 300 mM NaCl, 1% TX-100). Pooled Flag eluates were then used for in vitro deubiquitylation experiments using either a Ubiquitin Chain Restriction (UbiCRest) kit (Boston Biochem, K-400) or recombinant GST-USP9X (Boston Biochem, E-552). The reaction was performed in a final volume of 25 μl for 30 minutes at 37°C and stopped by addition of 5 μl of 5× sample buffer and boiling the samples at 95°C for 5 minutes. The samples were resolved as above.

### c-MYC transcriptional activity.

MYC transcriptional activity was determined in HCT116 cells using a Cignal Myc Reporter Assay kit from SABiosciences (CCS-012L).

### Mouse lines.

Mice carrying conditional alleles for *Usp9x* and *Fbw7* were previously described (16, 33). Similarly, *c-Jun^fl/fl^* and *Villin-Cre* mice were described before (41, 42). *c-Myc^fl/fl^* mice were a gift from Dinis Calado (The Francis Crick Institute).

### Histological analysis and quantifications.

Mice injected with 100 mg/kg BrdU (MilliporeSigma) i.p. 2.5 hours prior to sacrifice were euthanized by cervical dislocation, and small intestines were dissected out. Intestines were cut longitudinally into pieces of similar size, opened, and fixed overnight in 10% neutral buffered formalin, briefly washed with PBS and transferred into 70% ethanol, roll processed, and embedded in paraffin. Sections were cut at 4 μm for H&E staining, AB/PAS staining, IHC, and immunofluorescence. Antibodies against Usp9x (Bethyl Laboratories, catalog A301-351), activated Notch1 (Abcam, ab8925), c-Myc (Santa Cruz Biotechnology Inc., sc-788), and lysozyme (Dako, A0099) were used.

For quantification of the average BrdU^+^ cells per crypt, 100 full crypts were scored from 3–8 mice per group. Goblet cells were quantified from 100 ileal villi from at least 5 mice per group. Paneth cells were quantified from 100 crypts from at least 5 mice per group.

### DBZ treatment.

Six- to- 8-week-old mice were treated with 2 mg/kg body weight γ-secretase inhibitor (DBZ; Tocris, catalog 4489) i.p. for 5 consecutive days. All mice were injected with 100 mg/kg BrdU 4 hours after the final DBZ injection and culled 2–3 hours after BrdU injection. The gut rolls were prepared as described above.

### DSS-induced colitis.

Mice were fed 2.5% DSS in drinking water for 7 days. At day 11 and/or 21, all mice were culled 2.5 hours after 100 mg/kg body weight BrdU injection. Colons were washed in ice-cold PBS and fixed in 10% neutral buffered formalin overnight. Fixed tissues were processed for IHC as described above.

### AOM/DSS-induced CRC.

Mice were given a single i.p. injection of 10 mg/kg body weight AOM (MilliporeSigma). One week after AOM injection, mice were fed 2.5% DSS in drinking water for another week. All mice were humanely killed either 12 weeks after AOM injection or when they showed signs of ill health.

### Survival analysis.

The Cancer Genome Atlas (TCGA) RNASeqv2 RSEM gene abundance estimates for 382 colorectal adenocarcinoma patient tumor samples were downloaded from cBioPortal (43, 44) and combined with patients’ clinical information. Samples with missing overall survival status/time were removed from the analysis. Those remaining were stratified into either low- or high-USP9X expression groups based on a 2/3 split of the abundance scores for that gene. A Kaplan-Meier survival curve was generated, and a log-rank test was used to determine the significance of the difference in overall survival due to the expression groups.

### Mutational analysis of CRC cohorts.

Data for 831 exome-sequenced colorectal adenocarcinoma patient samples with corresponding sex information were downloaded from cBioPortal (42) to test for co-occurrence of *USP9X* and *FBW7* mutations using a Fisher’s exact test. The samples were composed of 2 cohorts: TCGA dataset (*n* = 212) (6) and the Dana-Farber Cancer Institute (DFCI) dataset (*n* = 619) (45).

### Statistics.

Statistical analysis was carried out using GraphPad Prism software. Two-tailed Student’s *t* tests were used to generate *P* values, except for Figure 5C and Figure 6, C and D, where 1-way ANOVA was used. A *P* value less than 0.05 was considered significant.

### Study approval.

Mouse experiments were carried out in accordance with UK Home Office guidelines and with the approval of the Animal Welfare and Ethical Review Body of the Francis Crick Institute.

## Author contributions

AB and OMK designed the experiments and wrote the manuscript. OMK performed the majority of experiments and analyzed the data. APS and DF performed the FBW7 MS experiment and analyzed the data. BSD and JC performed IHC and RNAscope experiments. RM performed survival analysis. SAW provided the *Usp9x*-conditional mouse.

## Supplementary Material

Supplemental data

## Version 1 01/18/2018

In-Press Preview

## Version 2 02/26/2018

Electronic publication

## Version 3 04/02/2018

Print issue publication
