# Supplementary material for: The deubiquitinase USP9X regulates FBW7 stability and suppresses colorectal cancer
Source: J Clin Invest. 2018 Feb 26;128(4):1326–37. doi: 10.1172/JCI97325 (PMC5873885; doi:10.1172/JCI97325)
Supplement: Supplemental data [file jci-128-97325-s001.pdf]

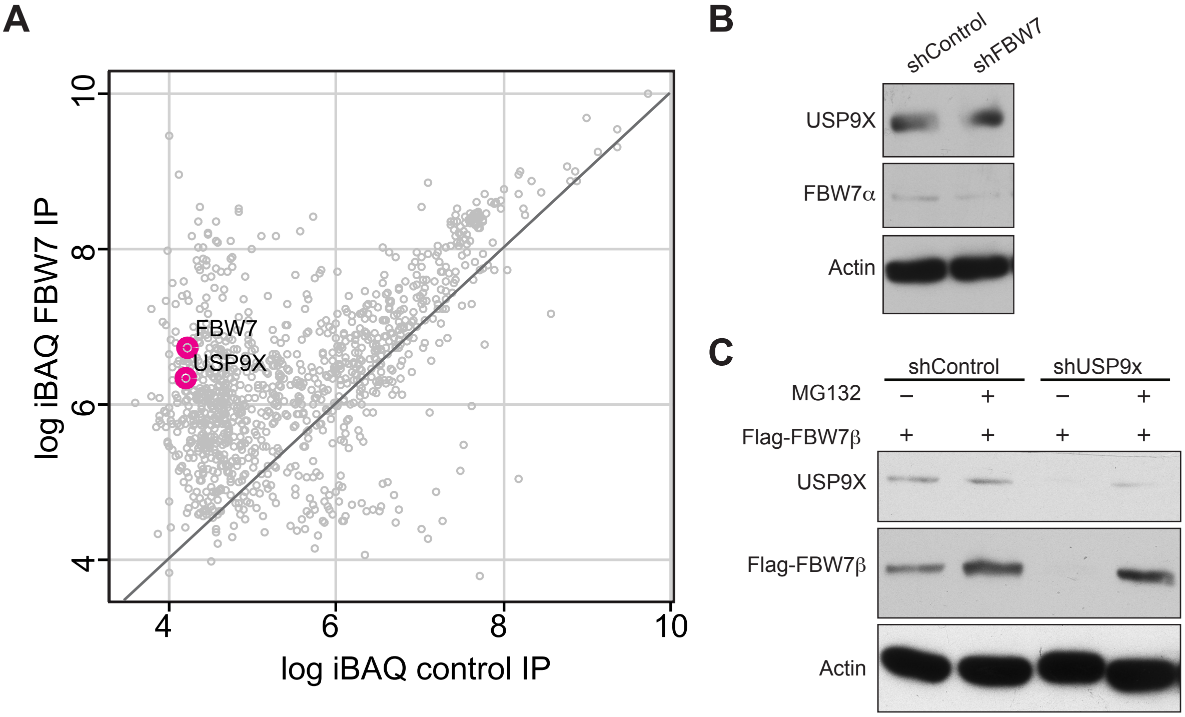

Supplementary Figure 1. (**A**) iBAQ plot showing MS-enrichment of FBW7 and interaction partners over a control IgG. (**B**) Western blots for indicated proteins in cells transfected with indicated shRNAs. (**C**) Western blots for indicated proteins in cells co-transfected with Flag-FBW7β and indicated shRNAs.

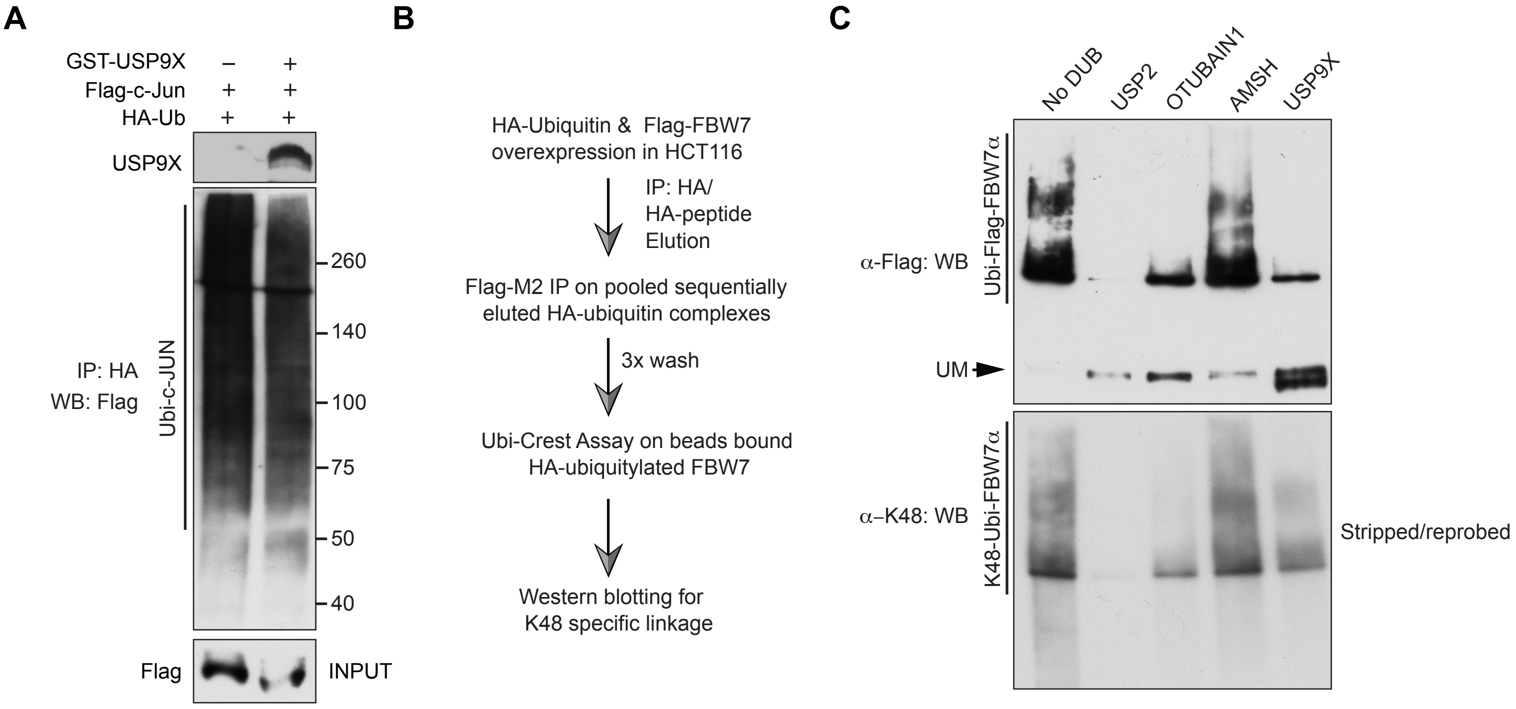

Supplementary Figure 2. USP9X cleaves K48-linked polyubiquitin chains on FBW7.
(**A**) Recombinant USP9X does not affect polyubiquitination of c-JUN in an in vitro deubiquitylation reaction. (**B**) Schematic for experiment in **C**. (**C**) Ubiquitin chain restriction (UbiCRest) experiment with indicated deubiquitinases: USP2 (promiscuous), OTUBAIN1 (K48-linked), and AMSH (K63-linked).

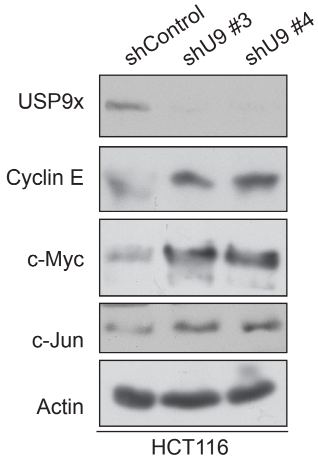

Supplementary Figure 3. Accumulation of SCF(FBW7) substrates in cells transfected with *USP9X*-shRNA compared to a non-targeting control.

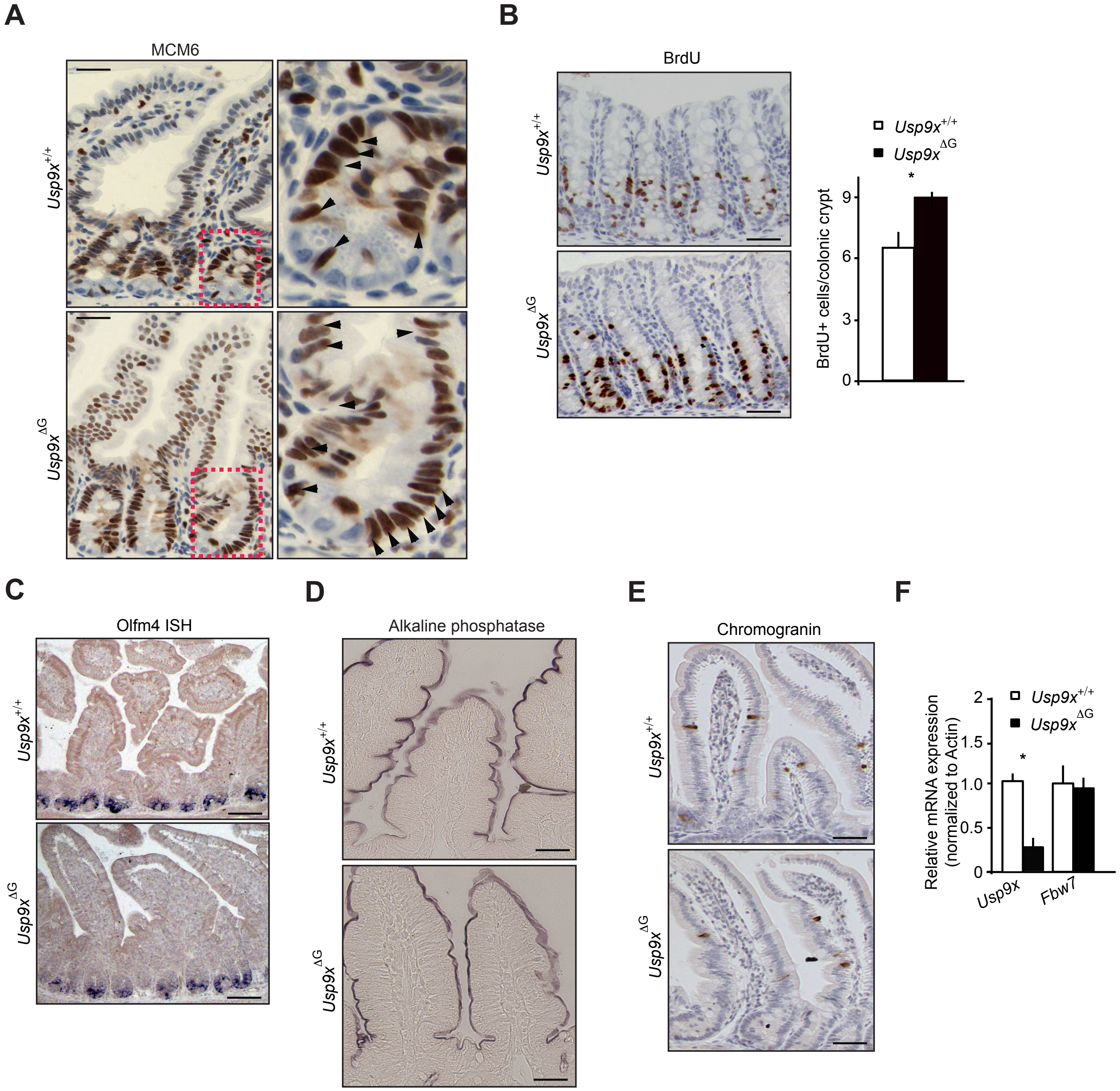

Supplementary Figure 4. (**A**) IHC sections stained for proliferating cells (MCM6) from the intestine of indicated mice. Scale bar = 50 µm. (**B**) BrdU staining for proliferating cells in colonic crypts from indicated mice, quantification shown in right panel. Scale bar = 100 µm, *n* = 3–4 mice/group. (**C**) *In situ* hybridization for *Olfm4* (stem cells) on the sections from the intestine of indicated mice. Scale bar = 100 µm. (**D** and **E**) IHC for enterocytes (Alkaline phosphatase) and enteroendocrine cells (Chromogranin) in gut from indicated mice. Scale bars = 100 µm. (**F**) qRT-PCR analysis showing mRNA levels of indicated genes normalised to actin and represented as fold change over control, in isolated crypts from *Usp9x*$^{+/+}$ (wildtype) and *Usp9x*$^{\Delta G}$ gut, *n* = 5–8 mice/group.

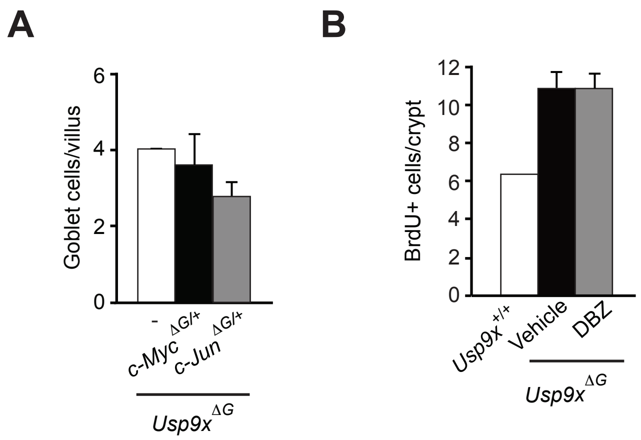

Supplementary Figure 5. (**A**) Average goblet cell number per villus from indicated mice. (**B**) Average BrdU+ cell number per crypt from indicated mice.

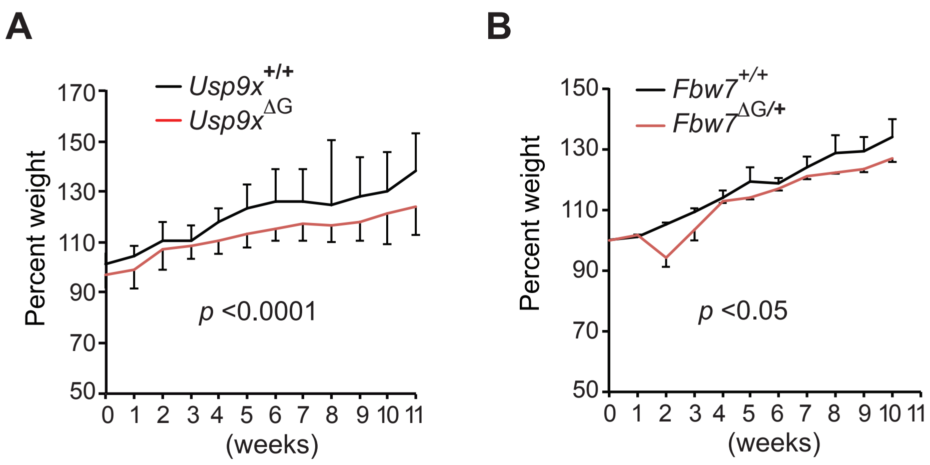

Supplementary Figure 6. (**A** and **B**) Weight curves presented as percent of starting weight in indicated mice from colitis-driven tumorigenesis experiment, n = 5–9 animals/genotype. P values were calculated by Log-rank (Mantel-Cox) test.

Supplementary Table. Shortlisted candidates from IP-mass spectrometry experiment using endogenous FBW7 as bait.

Gene Symbol	Description	Function
**FBXW7**	**F-box/WD repeat-containing protein 7**	**E3 Ligase**
**SKP1**	**S-phase kinase-associated protein 1**	**SCF Component**
UBAP2L	Ubiquitin-associated protein 2-like	Ubiquitylation
MYCBP2	MYC binding protein 2	Probable E3 ubiquitin-protein ligase
HERC2	E3 ubiquitin-protein ligase HERC2	E3 Ligase
HUWE1	E3 ubiquitin-protein ligase HUWE1	E3 Ligase
HECTD1	E3 ubiquitin-protein ligase HECTD1	E3 Ligase
**USP9X**	**Probable ubiquitin carboxyl-terminal hydrolase FAF-X**	**Deubiquitinase**
USP20	Ubiquitin carboxyl-terminal hydrolase 20	Deubiquitinase
FKBP8	Peptidyl-prolyl cis-trans isomerase FKBP8	Chaperon
FKBP4	Peptidyl-prolyl cis-trans isomerase FKBP4	Chaperon
PSMA2	Proteasome subunit alpha type-2	Proteasome subunit
PSMA3	Proteasome subunit alpha type-3	Proteasome subunit
PSMA6	Proteasome subunit alpha type	Proteasome subunit
PSMD3	26S proteasome non-ATPase regulatory subunit 3	Proteasome subunit
FOXP2	Forkhead box protein P2	Transcription factor
c-JUN	Transcription factor AP-1	Transcription factor
STAT1	Signal transducer and activator of transcription 1-alpha/beta	Transcription factor
MSH6	DNA mismatch repair protein Msh6	DNA replication/Genome integrity
MCM3	DNA replication licensing factor MCM3	DNA replication/Genome integrity
MCM7	DNA replication licensing factor MCM7	DNA replication/Genome integrity
TOP1	DNA topoisomerase 1	DNA replication/Genome integrity
RAD21	Double-strand-break repair protein rad21 homolog	DNA replication/Genome integrity
NAP1L1	Nucleosome assembly protein 1-like 1	DNA replication/Genome integrity
HDAC2	Histone deacetylase 2;Histone deacetylase	DNA replication/Genome integrity
CHD4	Chromodomain-helicase-DNA-binding protein 4	DNA replication/Genome integrity
MRE11A	Double-strand break repair protein MRE11A	DNA replication/Genome integrity
DNAJB6	DnaJ homolog subfamily B member 6	DNA replication/Genome integrity
MTOR	Serine/threonine-protein kinase mTOR	Kinase
STK3	Serine/threonine-protein kinase 3	Kinase
PANK2	Pantothenate kinase 2, mitochondrial	Kinase
GALK1	Galactokinase	Kinase
DEK	Protein DEK	Kinase
CSNK1A1	Casein kinase I isoform alpha	Kinase
PI4KB	Phosphatidylinositol 4-kinase beta	Kinase
WEE1	Wee1-like protein kinase	Kinase
XPO1	Exportin-1	Nuclear export
CSE1L	Exportin-2	Nuclear export
KPNB1	Importin subunit beta-1	Nuclear import
RANBP1	Ran-specific GTPase-activating protein	Transport
LUC7L	Putative RNA-binding protein Luc7-like 1	RNA binding/processing

DDX39	ATP-dependent RNA helicase DDX39A	RNA binding/processing
MARS	Methionine-tRNA ligase, cytoplasmic	RNA binding/processing
RAE1	mRNA export factor	RNA binding/processing
BCAS2	Pre-mRNA-splicing factor SPF27	RNA binding/processing
LARS	Leucine--tRNA ligase, cytoplasmic	RNA binding/processing
RBM39	RNA-binding protein 39	RNA binding/processing
RBM28	RNA-binding protein 28	RNA binding/processing
DDX24	ATP-dependent RNA helicase DDX24	RNA binding/processing
TARS	Threonine--tRNA ligase, cytoplasmic	RNA binding/processing
FMR1	Fragile X mental retardation protein 1	RNA binding/processing
IARS	Isoleucine--tRNA ligase, cytoplasmic	RNA binding/processing
YBX2	DNA-binding protein A;Y-box-binding protein 2	RNA binding/processing
MLL	Histone-lysine N-methyltransferase	Enzyme
RPN2	DPD glycosyltransferase subunit 2	Enzyme
DPM1	Dolichol-phosphate mannosyltransferase	Enzyme
PRMT1	Protein arginine N-methyltransferase 1	Enzyme
ACLY	ATP-citrate synthase	Enzyme
EIF4G1	Eukaryotic translation initiation factor 4 gamma 1	Translation
EIF3F	Eukaryotic translation initiation factor 3 subunit F	Translation
EIF2B4	Translation initiation factor eIF-2B subunit delta	Translation
EIF5B	Eukaryotic translation initiation factor 5B	Translation
MFF	Mitochondrial fission factor	Mitochondrial
SSBP1	Single-stranded DNA-binding protein, mitochondrial	Mitochondrial
ATP5J2	ATP synthase subunit f, mitochondrial	Mitochondrial
MTCH2	Mitochondrial carrier homolog 2	Mitochondrial
MAGED2	Melanoma-associated antigen D2	Cell adhesion
ACTR2	Actin-related protein 2	Cytoskeleton
TUBA1C	Tubulin alpha-1C chain	Cytoskeleton
TUBB2A	Tubulin beta-2A chain	Cytoskeleton
